# In vitro activity of seven antifungal agents against *Fusarium oxysporum* and expression of related regulatory genes

**DOI:** 10.1371/journal.pone.0322206

**Published:** 2025-04-29

**Authors:** Dafeng Xu, Kun Wang, Tingchun Li, Jingjing Wang, Shiji Wang, Fanna Kong, Jie Dai, Yuguo Liu, Banfeng Ruan, Benguo Zhou

**Affiliations:** 1 Institute of Industrial Crops, Anhui Academy of Agricultural Sciences, Hefei, China; 2 School of Biological Food and Environment, Hefei University, Hefei, China; 3 Huainan Academy of Agricultural Sciences, Huainan, China; 4 Hachikō Legend Culture & Tourism Group, Huainan, China; ICAR - Indian Agricultural Research Institute, INDIA

## Abstract

*Fusarium oxysporum* (*F. oxysporum*) is one of the main pathogenic fungus causing maize ear rot. In this study, the aims were to screen highly effective pesticides for *F. oxysporum*, reduce peasants’ misunderstandings about pesticide application, improve disease control levels, and enhance economic efficiency. The toxicity of seven fungicides (carbendazim, pyraclostrobin, epoxiconazole, tricyclazole, azoxystrobin, difenoconazole, quintozene) on *F. oxysporum* were determined by the mycelium growth rate and the spore germination method, and single and compound fungicides with effective inhibitory effects on mycelial growth were screened. The RT-qPCR method was used to detect the expression levels of chitin synthetase V (*ChsV*), folate uptake block T (*FUBT*), superoxide dismutase (*SOD*), and peroxidase dismutase (*POD*) genes in pathogenic bacteria treated with the selected agents and combination of fungicides. The results showed that all seven fungicides had inhibitory effects on mycelial growth hyphae and spore germination of *F. oxysporum*. Epoxiconazole had the strongest inhibitory effect on mycelium growth and spore germination of *F. oxysporum*, with effective concentrations (EC_50_) of 0.047 and 0.088 μg/mL, respectively. The combination of pyraclostrobin and difenoconazole (P&D, combined at a mass ratio of 7:3) had the best inhibitory effect, with an EC_50_ of 0.094 μg/mL and an SR of 2.650. Epoxiconazole and the combination P&D could inhibit mycelial growth and spore germination by down-regulating *ChsV*, *FUBT*, and *POD*, causing oxidative stress in *F. oxysporum*, and reducing the occurrence of maize ear rot.

## 1. Introduction

Maize ear rot, a prevalent and highly damaging fungal disease worldwide, significantly impacts maize yield and quality, and it brings great safety hazards to food and feed [1,2]. Changes such as the replacement of maize varieties, increased planting density, and alterations in cultivation practices have created favorable conditions for the occurrence and prevalence of maize ear rot [3,4]. This disease is caused by various fungal infections, with *Fusarium graminearum* (*F*. *graminearum*), *Fusarium oxysporum* (*F*. *oxysporum*), and *Fusarium verticillioides* (*F*. *verticillioides*) being the major pathogens [5,6]. Moreover, all three mentioned *Fusarium* fungi can produce fungal toxins that are associated with various diseases in both humans and animals [3,7]. *Fusarium* spp. can infect maize ears and grains, producing fungal toxins, a process regulated by related genes. The folate uptake block T gene (*FUBT*) can regulate the production of fusaric acid (FA) by *F*. *oxysporum* [8]. The chitin synthase gene (Chitin synthetase V, *ChsV*) can protect *Fusarium* from plant antimicrobial substances, and the absence of the *ChsV* gene leads to a loss of its pathogenicity [9]. *Fusarium* spp. itself possesses numerous protective enzyme genes, such as the superoxide dismutase (*SOD*) gene and the peroxidase dismutase (*POD*) gene [10]. SOD is a crucial enzyme in the antioxidant system, playing a central role in the elimination of reactive oxygen species [11], which helps defend *Fusarium* spp. from oxygen toxicity and oxidative damage. Under stress conditions, *POD* can efficiently eliminate H_2_O_2_, and its activity can reflect the metabolic status of the organism and its adaptability to the environment [12]. FA is a non-specific toxin produced by *F*. *oxysporum*, which can reduce host resistance by altering the permeability of host plant cell membranes, decreasing mitochondrial reactive oxygen species, inhibiting ATP synthesis, and suppressing plant root growth [13,14]. At present, research on *FUBT* and *ChsV* has made some progress in the fusarium wilt of watermelon, melon, and cotton caused by *F. oxysporum*, but there are few reports on maize ear rot [15,16].

As people’s awareness of health increases, there is a growing concern about fungal toxin contamination in maize. Addressing and preventing maize ear rot disease and reducing fungal toxin contamination has become a current research focus. Currently, there are few varieties resistant to maize ear rot, and biological control methods are not yet mature [17,18]; chemical control is the most widely applied measure [19]. Fungicides can effectively control the occurrence and spread of maize ear rot in the short term, significantly reducing the content of toxins in the kernels [18]. However, there are a wide variety of fungicides on the market, each with different chemical structures and mechanisms of action. Improper use can easily lead to phytotoxicity issues. Therefore, conducting toxicity tests for different fungicides on maize ear rot is the primary task to ensure the selection of appropriate agents. In order to reduce the dosage and frequency of fungicide use, delay the development of resistance, and minimize phytotoxicity, it is essential to develop rational combinations of fungicides. This study focused on the toxicity and synergistic effects of seven commonly used fungicides for maize ear rot on *F*. *oxysporum*. The best single and compound fungicides were screened, and their effects on the expression levels of *ChsV*, *FUBT*, *SOD*, and *POD* genes in *F. oxysporum* are further analyzed, providing reference points for the effective control of maize ear rot.

## 2. Materials and methods

### 2.1 Materials

*Fusarium oxysporum* B (*F*. *oxysporum* B) was provided by the Plant Protection Laboratory of the Tobacco Research Institute, Anhui Academy of Agricultural Sciences. The potato dextrose agar medium (PDA), potato glucose broth medium (PDB), and water agar medium (WA) were prepared following the method outlined by Fang Zhongda [20].

Fungicides: Carbendazim (95%, Shandong Huayang pesticide chemical industry group Co., Ltd.), Pyraclostrobin (98%, Anhui Kelihua Chemical Co., Ltd.), Epoxiconazole (98%, Ningxia Gree Fine Chemical Co., Ltd.), Tricyclazole (95%, Shandong Shangnong Agricultural Technology Co., Ltd.), Azoxystrobin (96%, Shandong Union Pesticide Industry Co., Ltd.), Difenoconazole (97%, Jiangsu Heben Biochemical Co., Ltd.), Quintozene (40%, Shanxi Nongfengbao Pesticide Co., Ltd.).

### 2.2 Toxicity determination of fungicides against *F. oxysporum* B

#### 2.2.1 Action of a single fungicide on the mycelium of *F. oxysporum* B.

The impact of different fungicides on the mycelial growth of *F. oxysporum* B was determined by the growth rate method [21]. Specifically, according to the method described by Kowalska Krochmal et al. [22], the minimum inhibitory concentration test was conducted to prepare PDA plates containing different doses (as shown in Table 1) of fungicides. A mycelial disc with a diameter of 6 mm was inoculated at the center of each plate with *F. oxysporum* B. Then the plates were cultivated in the dark at 25°C with three replicates for each treatment, and PDA plates without any fungicide served as the control. After 3 days, the colony diameter was measured, and the mycelial growth inhibition rate (MGIR) was calculated according to formula (1).

**Table 1:** Mass concentration of fungicides for inhibiting mycelial growth.

Fungicide	Mass concentration (μg/mL)				
Carbendazim	0.30	0.40	0.50	0.60	0.70
Pyraclostrobin	0.10	1	10	100	500
Epoxiconazole	0.0125	0.025	0.05	0.10	0.20
Tricyclazole	6.25	12.50	25.00	50	100
Azoxystrobin	4.00	8.00	16.00	32.00	64.00
Difenoconazole	0.01	0.05	0.10	0.50	1.00
Quintozene	3.125	6.25	12.50	25	50


$$MGIR=\frac{\varphi_{c}-\varphi_{t}}{\varphi_{t}-6}\times 100\%$$ (1)

Where: MGIR, mycelial growth inhibition rate; φ_c_, colony diameter in control group; φ_t_, colony diameter in the treatment group; 6, colony diameter of the initial mycelial disc.

The MGIR were converted into probability values of inhibition rate; the logarithm of the fungicide mass concentration was used as the horizontal axis, and the probability values were used as the vertical axis to fit the regression equation. Then the correlation coefficient (R^2^) and effective concentration (EC_50_) were calculated, and the toxicity levels of 7 fungicides against the mycelium of *F. oxysporum* B were compared.

#### 2.2.2 Action of a single fungicide on the spore of *F. oxysporum* B.

The impact of different fungicides at a single dose on the germination of spores of *F*. *oxysporum* B was determined by the spread plate method [23]. Specifically, *F. oxysporum* B was cultured on PDA plates for 3 days. The mycelium was rinsed with sterile water, filtered through double layers of sterile gauze, and the filtrate was centrifuged at 4,000 rpm for 10 min. Then the spore was resuspended in sterile deionized water to prepare a spore suspension with a concentration of 10^6^ spores/mL. 100 μL of spore suspension was spread on WA plates containing different doses (as shown in Table 2) of fungicides and incubated at 25°C in the dark. With three replicates for each treatment, and WA plates without any fungicide served as the control. When the spore germination rate on the control WA plate reached 90% or more, the number of germinated spores was recorded for different mass concentrations of the fungicide treatment. The spore germination rate (SGR) and spore germination inhibition rate (SGIR) were calculated according to formulas (2) and (3), respectively.

**Table 2:** Mass concentration of fungicides for inhibiting spore germination.

Fungicide	Mass concentration (μg/mL)				
Carbendazim	2	4	8	16	32
Pyraclostrobin	0.125	0.25	0.50	1	2
Epoxiconazole	0.05	0.10	0.20	0.40	0.80
Tricyclazole	12.50	25	50	100	200
Azoxystrobin	4	8	16	32	64
Difenoconazole	2	4	8	16	32
Quintozene	3.125	6.25	12.50	25	50


$$SGR=\frac{n_{t}}{n_{0}}\times 100\%$$ (2)

$$SGIR=\frac{{SGR}_{c}-{SGR}_{t}}{{SGR}_{c}}\times 100\%$$ (3)

Where: SGR, spore germination rate; n_t_, the number of germinated spores; n_0_, the total number of spores; SGIR, spore germination inhibition rate; SGIR_c_, SGR of the control group; SGIR_t_, SGR of the treatment group.

The SGIR were converted into probability values of inhibition rate; the logarithm of the fungicide mass concentration was used as the horizontal axis, and the probability values were used as the vertical axis to fit the regression equation. Then the R^2^ and EC_50_ were calculated, and the toxicity levels of 7 fungicides against the spore of *F*. *oxysporum* B were compared.

#### 2.2.3 Action of compound fungicides on the mycelium of *F. oxysporum* B.

Based on the measurement results of 2.2.1 and 2.2.2, compound the fungicide. The effects of compound fungicides were determined by the growth rate method [21]. Specifically, dilute each fungicide separately to 100 μg/mL, and then prepare different proportions of mixed solutions according to Table 3, and prepare PDA plates containing 10% compound fungicide solution. The procedures outlined in Section 2.2.1 were repeated, *F. oxysporum* B mycelial disc (whose diameter was 6 mm) was inoculated, and the MGIR for the composite fungicide was calculated. Then we fitted a regression equation, and the R2 and EC_50_ were calculated. Analyzing the synergistic enhancement effect of the combination agent based on the method proposed by Wadley [24,25], using the synergistic ratio (SR) for the analysis of combined enhancement effects (Formulas 4 and 5), SR<0.5 indicates antagonistic effects in the compound formulation of the two fungicides; 0.5≤SR≤1.5 indicates additive effects in the compound formulation of the two fungicides; SR>1.5 indicates synergistic effects in the compound formulation of the two fungicides.

**Table 3:** Mass concentration of composite agents used to determine the inhibitory effect of mycelium growth.

Mass ratio	Mass concentration (μg/mL)			
	Epoxiconazole: Carbendazim	Carbendazim: Quintozene	Pyraclostrobin: Difenoconazole	Pyraclostrobin: Carbendazim
1:9	0.05, 0.1, 0.2, 0.3, 0.4	1.8, 2.4, 3.0, 3.6, 4.2	0.03125, 0.0625, 0.125, 0.25, 0.5	0.1, 0.2, 0.4, 0.6, 0.8
2:8	0.05, 0.1, 0.2, 0.3, 0.4	0.6, 1.2, 1.8, 2.4, 3.0	0.03125, 0.0625, 0.125, 0.25, 0.5	0.1, 0.2, 0.4, 0.6, 0.8
3:7	0.05, 0.1, 0.2, 0.3, 0.4	0.6, 1.2, 1.8, 2.4, 3.0	0.125, 0.25, 0.5, 1, 2	0.2, 0.3, 0.4, 0.5, 0.6
4:6	0.025, 0.05, 0.1, 0.2, 0.3	0.4, 0.6, 0.8, 1.0, 1.2	0.125, 0.25, 0.5, 1, 2	0.4, 0.5, 0.6, 0.7, 0.8
5:5	0.025, 0.05, 0.1, 0.2, 0.3	0.4, 0.6, 0.8, 1.0, 1.2	0.03125, 0.0625, 0.125, 0.25, 0.5	0.4, 0.5, 0.6, 0.7, 0.8
6:4	0.025, 0.05, 0.1, 0.2, 0.4	0.4, 0.5, 0.6, 0.7, 0.8	0.0625, 0.125, 0.25, 0.5, 1	0.4, 0.6, 0.8, 1.0, 1.2
7:3	0.025, 0.05, 0.1, 0.2, 0.4	0.3, 0.4, 0.5, 0.6, 0.7	0.0625, 0.125, 0.25, 0.5, 1	0.4, 0.6, 0.8, 1.0, 1.2
8:2	0.0125, 0.025, 0.05, 0.1, 0.2	0.4, 0.5, 0.6, 0.7, 0.8	0.0625, 0.125, 0.25, 0.5, 1	0.4, 0.6, 0.8, 1.0, 1.2
9:1	0.0125, 0.025, 0.05, 0.1, 0.2	0.3, 0.4, 0.5, 0.6, 0.7	0.0625, 0.125, 0.25, 0.5, 1	0.25, 0.5, 1.0, 2.0, 3.0


$$SR=\frac{{Theory\;EC}_{50}}{{Actual\;EC}_{50}}$$ (4)

$${Theory\;EC}_{50}=\frac{a+b}{\frac{a}{{EC}_{50A}}+\frac{b}{{EC}_{50B}}}$$ (5)

Where: SR, synergistic ratio; A (or B): one type of fungicide; a (or b): the mass (or volume) ratio of fungicide A (or B).

### 2.3 mRNA expression analysis

A single fungicide and a compound agent were selected that have inhibitory or synergistic effects on *F. oxysporum* B. Prepare PDA plates containing a fungicide or compound agent and cultivate *F. oxysporum* B; and the normal PDA plate was used as the control group. After 3 days, mycelium was collected. The mRNA levels of *ChsV* (ChsV-F: 5’-TCTTTTCCCCATCAAGTGTCT-3’; ChsV-R: 5’-GTGATGTTGGTGTTTCCGGTTGT-3’), *FUBT* (FUBT-F: 5’-GGAGCCTGAAGACAGATTGC-3’; FUBT-R:5’-CCGATAATAGGGACGATCCA-3’), *SOD* (SOD-F: 5’-GGTCCTCACTTCAACCCTCA-3’; SOD-R: 5’-AGTCGGTGACAGAGCCCTTA-3’), *POD* (POD-F: 5’-CGAGGGATGGATCAAGGATA-3’; POD-R: 5’-GTAGCATCCTGCTGGTCGAT-3’) [16,26] in the mycelium of *F. oxysporum* B were measured. The TRIzol (TIANGEN Biotech (Beijing) Co., Ltd.) method is utilized to extract mRNA from the mycelium. Then a 1μg mRNA of satisfactory quality is selected for reverse transcription to obtain cDNA (ReverTra Ace qPCR RT Master Mix with gDNA Remover; TOYOBO Co., Ltd.), which is subsequently subjected to qPCR. The relative mRNA abundance of the target genes was normalized to *Actin* (Actin-F: 5’-CCGTGACATCAAGGAGAAGC-3’; Actin-R: 5’-GGAAAGTGGACAGGGAAGCA-3’) and was then calculated using the 2^−ΔΔCt^ method.

### 2.4 Statistical analysis

Microsoft Excel 2010 software was used to process the data. Statistical analysis was conducted using SPSS 19.0 (SPSS, Inc., Chicago, USA). Significant differences were obtained by one-way ANOVA, and the difference was considered significant when *P* ≤ 0.05.

## 3 Results

### 3.1 Inhibitory effect of a single fungicide on the mycelial growth of *F. oxysporum* B

The effects of a single fungicide on mycelial growth are shown in Table 4. It can be observed that the mycelial growth of *F. oxysporum* B is inhibited by all seven tested fungicides; the EC_50_ is 0.047~35.089 μg/mL. And the three fungicides with the strongest inhibitory effect on mycelial growth are epoxiconazole, difenoconazole, and carbendazim. Their EC_50_ are as follows: 0.047 μg/mL, 0.078 μg/mL, and 0.445 μg/mL. The inhibitory effects of azoxystrobin on mycelial growth are the weakest, with an EC_50_ of 35.089 μg/mL. These results indicate that epoxiconazole, difenoconazole, and carbendazim are effective in inhibiting the mycelial growth of *F. oxysporum* B.

**Table 4:** Toxicity test results of 7 fungicides in a single dose on the mycelial growth of *F*. *oxysporum* B.

Fungicide	Regression equation	EC_50_ (μg/mL)	95% confidence interval (μg/mL)	R^2^
Carbendazim	y=6.639x+7.338	0.445	0.424~0.464	0.988
Pyraclostrobin	y=0.364x+4.761	4.533	2.037~9.385	0.933
Epoxiconazole	y=1.173x+6.558	0.047	0.037~0.059	0.983
Tricyclazole	y=1.585x+2.589	33.172	27.881~40.047	0.983
Azoxystrobin	y=0.448x+4.308	35.089	19.627~139.043	0.973
Difenoconazole	y=0.818x+5.905	0.078	0.054~0.109	0.990
Quintozene	y=0.958x+4.256	5.978	3.954~8.027	0.990

### 3.2 Inhibitory effect of a single fungicide on the spore germination of *F. oxysporum* B

The effects of a single fungicide on spore germination are shown in Table 5. It can be observed that the mycelial growth of *F. oxysporum* B is inhibited by all seven tested fungicides; the EC_50_ is 0.088~42.720 μg/mL. And the two fungicides with the strongest inhibitory effect on mycelial growth are epoxiconazole and pyraclostrobin; their EC_50_ are as follows: 0.088 μg/mL, 0.249 μg/mL. The inhibitory effects of tricyclazole on mycelial growth are the weakest, with an EC_50_ of 42.720 μg/mL. These results indicate that epoxiconazole and pyraclostrobin are effective in inhibiting the spore germination of *F. oxysporum* B.

**Table 5:** Toxicity test results of 7 fungicides in a single dose on spore germination of *F*. *oxysporum* B.

Fungicide	Regression equation	EC_50_ (μg/mL)	95% confidence interval (μg/mL)	R^2^
Carbendazim	y=0.460x+4.431	17.285	9.813~62.681	0.970
Pyraclostrobin	y=1.184x+5.714	0.249	0.182~0.318	0.998
Epoxiconazole	y=0.327x+5.346	0.088	0.002~0.191	0.986
Tricyclazole	y=0.980x+3.402	42.720	31.869~55.890	0.953
Azoxystrobin	y=0.997x+3.573	26.963	20.610~30.005	0.935
Difenoconazole	y=1.417x+3.506	11.339	9.359~14.049	0.958
Quintozene	y=1.202x+3.487	18.142	14.102~22.641	0.960

### 3.3 Toxicity determination of compound fungicides on *F. oxysporum* B

#### 3.3.1 Inhibitory and synergistic effects of a mixture of epoxiconazole and carbendazim.

The range of EC_50_ values for a mixture of epoxiconazole and carbendazim with different ratios is 0.046 to 0.282 μg/mL, and the EC_50_ values of the mixture are all lower than the EC_50_ values of carbendazim (Table 6). The inhibitory effects of the mixtures are stronger than those of carbendazim. When the mass ratio of epoxiconazole and carbendazim is 8:2, the EC_50_ is 0.046 μg/mL, which is lower than the EC_50_ of epoxiconazole (0.047 μg/mL, 95% confidence interval: 0.037~0.059 μg/mL). At this mass ratio (8:2), the inhibitory effect is best. The SR values for a mixture of epoxiconazole and carbendazim at different mass ratios range from 0.413 to 1.236. When the mass ratio is 4:6 and 7:3, the SRs are 0.464 and 0.413 (SR<0.5), indicating an antagonistic effect. When the mass ratio is 1:9, 2:8, 3:7, 5:5, 6:4, 8:2, and 9:1, the SR are 0.855, 0.656, 0.921, 0.868, 1.159, 1.236, and 0.537, suggesting an additive effect (0.5≤SR≤1.5).

**Table 6:** Toxicity effects of the epoxiconazole and carbendazim mixtures on *F*. *oxysporum* B.

Fungicide combination	Mass ratio	Regression equation	R^2^	Actual EC_50_ (μg/mL)	95% confidence interval (μg/mL)	Theory EC_50_ (μg/mL)	SR
Epoxiconazole (A)	/	y=1.173x+1.558	0.983	0.047	0.037~0.059	/	/
Carbendazim (B)	/	y=6.639x+2.338	0.988	0.445	0.424~0.464	/	/
A+B	1:9	y=1.719x+5.946	0.993	0.282	0.203~0.286	0.241	0.855
A+B	2:8	y=1.291x+5.773	0.937	0.252	0.205~0.330	0.165	0.656
A+B	3:7	y=0.945x+5.817	0.939	0.136	0.098~0.180	0.126	0.921
A+B	4:6	y=0.944x+5.623	0.983	0.219	0.160~0.354	0.101	0.464
A+B	5:5	y=1.096x+6.105	0.977	0.098	0.077~0.126	0.085	0.868
A+B	6:4	y=0.963x+6.155	0.990	0.063	0.045~0.083	0.073	1.159
A+B	7:3	y=1.131x+0.915	0.981	0.155	0.122~0.207	0.064	0.413
A+B	8:2	y=1.170x+6.561	0.970	0.046	0.037~0.058	0.057	1.236
A+B	9:1	y=0.831x+5.846	0.990	0.096	0.069~0.154	0.052	0.537

Note: “/” indicates none or a value below 0.001

#### 3.3.2 Inhibitory and synergistic effects of a mixture of carbendazim and quintozene.

The EC_50_ values for a mixture of carbendazim and quintozene with different ratios is 0.437 to 3.684 μg/mL (Table 7), and all of the EC_50_ values for the mixture are lower than those for quintozene alone. Specifically, when the mass ratio of carbendazim and quintozene is 8:2, the EC_50_ is 0.437 μg/mL, which is lower than the EC_50_ of carbendazim (0.445 μg/mL, 95% confidence interval: 0.424~0.464 μg/mL). For all other combinations, the EC_50_ values for the mixtures are higher than that of carbendazim. However, the SR values for the mixtures of carbendazim and quintozene at various mass ratios range from 0.643 to 1.330, indicating an additive effect (0.5≤SR≤1.5).

**Table 7:** Toxicity effects of the carbendazim and quintozene mixtures on *F*. *oxysporum* B.

Fungicide combination	Mass ratio	Regression equation	R^2^	Actual EC_50_ (μg/mL)	95% confidence interval (μg/mL)	Theory EC_50_ (μg/mL)	SR
Carbendazim (A)	/	y=6.639x+2.338	0.988	0.445	0.424~0.464	/	/
Quintozene (B)	/	y=0.958x-0.744	0.990	5.978	3.954~8.027	/	/
A+B	1:9	y=3.081x+3.260	0.911	3.684	3.355~4.222	2.665	0.723
A+B	2:8	y=3.354x+4.324	0.968	1.591	1.290~1.930	1.714	1.078
A+B	3:7	y=3.165x+4.072	0.929	1.965	1.476~2.868	1.264	0.643
A+B	4:6	y=3.477x+4.919	0.979	1.055	0.965~1.187	1.001	0.949
A+B	5:5	y=4.915x+6.011	0.970	0.623	0.582~0.660	0.828	1.330
A+B	6:4	y=5.344x+5.647	0.986	0.757	0.711~0.825	0.707	0.934
A+B	7:3	y=4.599x+5.894	0.939	0.639	0.596~0.701	0.616	0.964
A+B	8:2	y=5.865x+7.111	0.968	0.437	0.402~0.464	0.546	1.251
A+B	9:1	y=4.409x+5.900	0.986	0.625	0.583~0.686	0.490	0.785

Note: “/” indicates none or a value below 0.001

#### 3.3.3 Inhibitory and synergistic effects of a mixture of pyraclostrobin and difenoconazole.

The EC_50_ values for a mixture of pyraclostrobin and difenoconazole at different mass ratios range from 0.044 to 0.176 μg/mL (Table 8). These values are significantly lower than the EC_50_ value for pyraclostrobin (4.533 μg/mL, 95% confidence interval: 2.037~9.385 μg/mL), indicating that the inhibitory effects of these combinations are significantly better than those of the single pyraclostrobin. When the mass ratio of pyraclostrobin and difenoconazole is 2:8, 3:7, 4:6, and 6:4, the EC_50_ values are 0.066 μg/mL, 0.044 μg/mL, 0.061 μg/mL, and 0.077 μg/mL. All these values are lower than the EC_50_ of a single propiconazole; this indicates that these four combinations have a better inhibitory effect on *F*. *oxysporum* B than a single pyraclostrobin. For combinations of mass ratios at 3:7, 4:6, 6:4, 7:3, 8:2, and 9:1, the SRs are 2.494, 2.120, 2.481, 2.650, 2.386, and 3.903, respectively, and these combinations indicate a clear synergistic effect (SR >1.5). Additionally, at mass ratios of 1:9, 2:8, and 5:5, the SR are 0.972, 1.474, and 0.873, respectively, suggesting an additive effect (0.5≤SR≤1.5). All SR values are greater than 0.5 in different mass ratios of pyraclostrobin and difenoconazole; these combinations indicate additive and significant synergistic effects, thus holding promise for practical application and further research.

**Table 8:** Toxicity effects of the pyraclostrobin and difenoconazole mixtures on *F*. *oxysporum* B.

Fungicide combination	Mass ratio	Regression equation	R^2^	Actual EC_50_ (μg/mL)	95% confidence interval (μg/mL)	Theory EC_50_ (μg/mL)	SR
Pyraclostrobin (A)	/	y=0.364x-0.239	0.933	4.533	2.037~9.385	/	/
Difenoconazole (B)	/	y=0.818x+0.905	0.990	0.078	0.054~0.109	/	/
A+B	1:9	y=0.680x+5.714	0.999	0.089	0.054~0.130	0.087	0.972
A+B	2:8	y=0.646x+5.763	0.941	0.066	0.034~0.099	0.097	1.474
A+B	3:7	y=0.646x+5.874	0.972	0.044	0.006~0.098	0.111	2.494
A+B	4:6	y=0.670x+5.815	0.987	0.061	0013~0.120	0.129	2.120
A+B	5:5	y=0.627x+5.474	0.966	0.176	0.101~0.265	0.153	0.873
A+B	6:4	y=0.677x+5.755	0.998	0.077	0.031~0.122	0.190	2.481
A+B	7:3	y=0.606x+5.621	0.969	0.094	0.038~0.151	0.250	2.650
A+B	8:2	y=0.661x+5.539	0.949	0.153	0.086~0.226	0.365	2.386
A+B	9:1	y=0.568x+5.434	0.970	0.173	0.091~0.502	0.675	3.903

Note: “/” indicates none or a value below 0.001

#### 3.3.4 Inhibitory and synergistic effects of a mixture of pyraclostrobin and carbendazim.

The EC50 values for a mixture of pyraclostrobin and carbendazim at different mass ratios range from 0.260 to 0.824 μg/mL (Table 9). And when the mass ratio is 1:9, 2:8, and 3:7, the EC_50_ values are 0.288 μg/mL, 0.260 μg/mL, and 0.379 μg/mL, all lower than the EC_50_ of single carbendazim (0.445 μg/mL, *P* ≤ 0.05). The mixture of pyraclostrobin and carbendazim, in the range of 1:9–3:7, has a better inhibitory effect on *F*. *oxysporum* B than a single carbendazim. When the mass ratio of pyraclostrobin to carbendazim is between 7:3 and 9:1, as the proportion of pyraclostrobin increases, the EC_50_ of the mixture gradually increases but remains significantly lower than the EC_50_ of the single pyraclostrobin (4.533 μg/mL, 95% confidence interval: 2.037~9.385 μg/mL). The SR gradually decreases as the mass ratio of pyraclostrobin to carbendazim changes from 2:8–5:5. And at the ratios of 4:6 and 5:5, the SR is 1.305 and 1.219, respectively, indicating an additive effect. However, in other mass ratios, SR is greater than 1.5, demonstrating a significant synergistic effect. All SR values are greater than 0.5 in different mass ratios of pyraclostrobin and carbendazim; these combinations indicate additive and significant synergistic effects. This combination formulation holds promise for practical application and further research.

**Table 9:** Toxicity effects of pyraclostrobin and carbendazim mixtures on *F*. *oxysporum* B.

Fungicide combination	Mass ratio	Regression equation	R^2^	Actual EC_50_ (μg/mL)	95% confidence interval (μg/mL)	Theory EC_50_ (μg/mL)	SR
Pyraclostrobin (A)	/	y=0.364x-0.239	0.933	4.533	2.037~9.385	/	/
Carbendazim (B)	/	y=6.639x+2.338	0.988	0.445	0.424~0.464	/	/
A+B	1:9	y=2.984x+6.614	0.961	0.288	0.204~0.386	0.241	0.855
A+B	2:8	y=2.312x+6.354	0.927	0.260,	0.148~0.391	0.165	0.656
A+B	3:7	y=2.500x+6.054	0.897	0.379	0.282~0.515	0.126	0.921
A+B	4:6	y=1.950x+5.532	0.957	0.533	0.435~0.610	0.101	0.464
A+B	5:5	y=1.357x+5.240	0.942	0.665	0.548~1.171	0.085	0.868
A+B	6:4	y=1.502x+5.413	0.927	0.531	0.377~0.638	0.073	1.159
A+B	7:3	y=1.257x+5.391	0.987	0.489	0.283~0.613	0.064	0.413
A+B	8:2	y=0.551x+5.078	0.887	0.721	/	0.057	1.236
A+B	9:1	y=0.363x+5.031	0.926	0.824	0.215~2.099	0.052	0.537

Note: “/” indicates none or a value below 0.001

### 3.4 Effect of fungicides on the expression of resistance genes in *F. oxysporum* B

**Fig 1:**
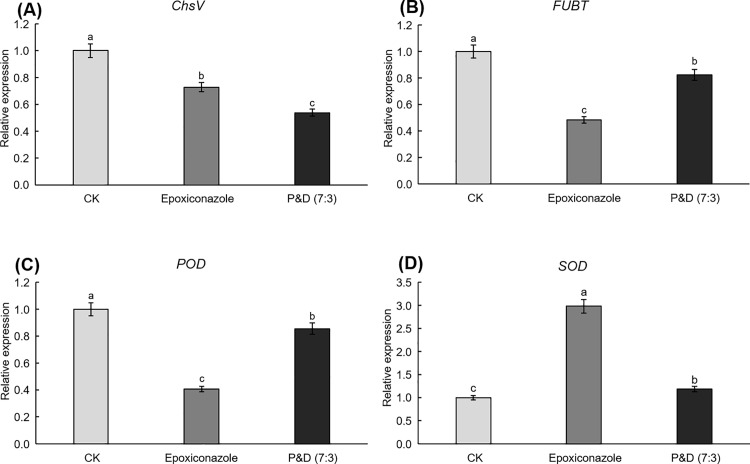
Effect of fungicides on the expression level of genes related to *F*. *oxysporum* B.

Based on the inhibitory effects of single fungicides and compound fungicides on *F*. *oxysporum* B, the expression levels of *ChsV*, *FUBT*, *SOD*, and *POD* in the epoxiconazole group, pyraclostrobin and difenoconazole (7:3) group (P&D (7:3)), and control group (CK) were determined using RT-qPCR. The results are shown in Fig 1. Compared to the CK, both epoxiconazole and P&D (7:3) treatments significantly downregulated the relative expression levels of *ChsV, FUBT*, and *POD* in *F*. *oxysporum* B. Additionally, the relative expression level of *SOD* was significantly increased in both treatments.

## 4. Discussion

Maize ear rot occurs in the ears of corn, making it difficult to control. In maize-growing regions such as the United States, Canada, and South Africa, the primary causative agent of ear rot is often Fusarium [27–29]. In most regions of China, such as Jilin, Anhui, and Heilongjiang, the primary causative agent of maize ear rot is also Fusarium [30–33]. At present, there are many reports on the toxicity determination of pathogenic fungi such as *F. graminearum* and *F*. *verticillioides* in maize ear rot, while there are fewer reports on *F*. *oxysporum* [34,35]. This study found that epoxiconazole exhibited the most effective inhibition of the mycelial growth and spore germination of *F*. *oxysporum*, followed by difenoconazole and pyraclostrobin. While individual fungicides showed inhibitory effects on *F*. *oxysporum*, long-term use of a single fungicide can easily lead to resistance to the fungus. Therefore, it is recommended to use compound formulations to mitigate this issue. This experiment revealed that the combination of pyrazoxystrobin and difenoconazole exhibited significant inhibition and synergistic effects on the mycelial growth of *F*. *oxysporum*. When the mass ratio of pyrazoxystrobin to difenoconazole was 7:3 (P&D (7:3)), the EC_50_ was 0.094 μg/mL, and the SR was 2.650.

Contains various pathogenic genes in *F*. *oxysporum* chitin synthase enzymes (Fochs). Among them, Fochs V and Fochs II play crucial roles in the pathogenicity of the strain [36]. Their absence results in a decrease in the pathogenic ability of the strain, and the loss of Fochs V leads to the loss of pathogenic capability in the strain [37]. Fusaric acid is a non-specific toxin that causes plant wilting. Research has demonstrated the presence of fusaric acid in cotton, leading to the occurrence of wilt disease. There is also evidence indicating a positive correlation between the virulence of *F*. *oxysporum* and the production of fusaric acid [13,14]. Additionally, *FUBT* has been shown to promote the production of fusaric acid. Knocking out the *FUBT* gene results in a significant reduction in fusaric acid production by *F*. *oxysporum* [8]. Antifungal agents can inhibit plant pathogens and cause damage by inducing the excessive production of reactive oxygen species (ROS) in the pathogens [38,39]. POD and SOD are critical enzymes in the reactive oxygen species (ROS) system. They have the ability to reduce or impede the damage caused by reactive oxygen-free radicals to organisms [40]. The activity of POD and SOD serves as important physiological indicators, reflecting the induced resistance of cells to the antifungal agent as well as their response to environmental stress [41,42]. Compared to the CK group, after treatment with epoxiconazole and P&D (7:3), the expression levels of *ChsV* and *FUBT* in *F*. *oxysporum* showed a significant decrease, suggesting a reduction in the pathogenicity and virulence of the fungus, which suggests a decreased likelihood of maize ear rot occurrence. And the expression level of the *SOD* exhibited an increasing trend, suggesting that the two treatments induced oxidative stress in *F*. *oxysporum*. Conversely, the expression level of the *POD* showed a significant decrease, indicating a reduction in the POD activity in *F*. *oxysporum*, leading to a weakened ability to eliminate H_2_O_2_. Therefore, the oxidative stress response in *F*. *oxysporum* was enhanced after treatment with epoxiconazole or P&D (7:3), leading to a decrease in its resistance to fungicides.

## 5. Conclusion

This study demonstrates the effectiveness of specific fungicides and their combinations in controlling *F. oxysporum*, a major pathogen causing maize ear rot. Epoxiconazole emerged as the most effective single fungicide, exhibiting strong inhibitory effects on both mycelial growth and spore germination. And compound formulations, particularly the combination of pyraclostrobin and difenoconazole (7:3 mass ratio), showed significant synergistic effects, providing superior control compared to individual fungicides. These fungicides downregulated key genes (*ChsV*, *FUBT*, and *POD*) and induced oxidative stress in *F. oxysporum*, reducing its environmental adaptability, infectivity, and pathogenicity. By optimizing fungicide combinations, this approach enhances disease control while minimizing pesticide use, offering a sustainable strategy to mitigate maize ear rot and its economic impact.
